# Seasonal Variation in Epidemiology of Kawasaki Disease-Related Coronary Artery Abnormalities in Japan, 1999–2017

**DOI:** 10.2188/jea.JE20190189

**Published:** 2021-02-05

**Authors:** Naomi Kitano, Takashi Takeuchi, Tomohiro Suenaga, Nobuyuki Kakimoto, Akihiro Naka, Shoichi Shibuta, Shinya Tachibana, Nobuhito Takekoshi, Takayuki Suzuki, Tomoya Tsuchihashi, Takashi Yamano, Takashi Akasaka, Hiroyuki Suzuki

**Affiliations:** 1 Research Center for Community Medicine, Wakayama Medical University, Wakayama, Japan; 2 Department of Public Health, Wakayama Medical University School of Medicine, Wakayama, Japan; 3 Department of Pediatrics, Wakayama Medical University School of Medicine, Wakayama, Japan; 4 Kinan Hospital, Wakayama, Japan; 5 Department of Cardiovascular Medicine, Wakayama Medical University School of Medicine, Wakayama, Japan

**Keywords:** coronary artery aneurysm, Kawasaki disease, patient age, seasonality, epidemiology

## Abstract

**Background:**

Epidemiological studies show a U-shaped tendency in Kawasaki disease (KD)-related coronary artery abnormalities (CAAs) across age categories. Since studies suggest seasonal variations in KD onset, this study aimed to clarify the epidemiologic features of CAAs, considering the seasons of KD-occurrence.

**Methods:**

We analyzed 2,106 (males = 1,215, females = 891) consecutive KD cases from October 1999 through September 2017 using our electronic database of annual surveys, targeting all hospitals with pediatric departments across Wakayama, Japan. The primary outcome was the presence/absence of CAAs measured by echocardiography 1 month after KD onset. Odds ratios (ORs) and 95% confidence intervals (CIs) of combined patient age and sex for CAAs were calculated using logistic regression models adjusted for four seasons.

**Results:**

The median age was 25 (range, 1–212) months. The proportion of males decreased with increasing age. The youngest age group (<6 months) showed an inverse summer/autumn to winter/spring ratio (>1.0) in KD-occurrence. CAAs were observed in 2.8% of cases (males = 3.4%, females = 2.1%), which significantly lessened in summer than in other seasons. Moreover, 50% (*n* = 4/8) of cases with giant aneurysms experienced KD in autumn. Adjusted ORs for CAAs among males aged ≥60 months (3.0; 95%, CI 1.2–7.5) and females aged <6 months (3.6; 95%, CI 1.1–11.8) were significantly higher than those among males aged 12–35 months.

**Conclusions:**

Cumulative 18-year data of consecutive KD cases from one area suggest the influence of interactions between patient age and sex on the development of KD-related CAAs. The season of KD-occurrence may reflect the diversity of agents.

## INTRODUCTION

Kawasaki disease (KD) is a medium-vessel systemic vasculitis that principally affects coronary arteries.^1^^–^^3^ Currently, KD is a leading cause of acquired heart disease in developed countries.^3^^–^^5^ Fifty years after the first 50 cases reported in 1967,^6^ the etiology of KD remains unknown. Japan has the highest incidence rate of KD worldwide.^7^^–^^9^ However, previous epidemiological studies have demonstrated seasonal variations of KD onset.^7^^,^^8^^,^^10^^–^^13^

KD mainly affects children younger than 5 years.^1^^,^^8^ As for the initial treatment in the acute phase of KD, the level of evidence regarding the efficacy of intravenous immunoglobulin (IVIG) treatment in preventing the development of KD-related coronary aneurysms is robust (first-line IVIG treatment: class Ia, grade A).^1^^,^^8^^,^^14^^–^^16^ However, the mechanisms underlying the therapeutic benefits of IVIG remain unclear. Moreover, the causes of KD remain unknown.^1^^,^^7^ Although IVIG is the established first-line treatment for KD, approximately 15–20% of all patients have persistent or recrudescent fever after receiving 2 g/kg of IVIG, and there has been considerable debate regarding the optimal second-line treatment in these patients.^1^^,^^16^

Previous epidemiological studies have demonstrated that younger^15^^,^^17^^–^^20^ and/or older^17^^,^^18^^,^^20^^,^^21^ age, as well as male sex,^15^^,^^17^^–^^20^ are associated with the development of coronary artery abnormalities (CAAs) in patients with acute-phase KD. The relationship between disease burden and age and the most vulnerable age categories in the development of KD-related CAAs have been identified; the representative graph forms a U-shaped curve with specific age groups (toddlers) at the bottom and other age groups in the arms.^17^^,^^18^^,^^20^^,^^22^ Our previous study, which included 17-year consecutive KD cases in one Japanese area, showed a sex difference in the distribution of males among age categories (oldest age group: male-to-female ratio <1).^23^ In the same paper, we also reported differing patterns in the seasonality of disease onset among age categories; KD occurred in summer and autumn in more than 60% of patients aged <4 months and in winter and spring in more than 60% of patients aged ≥7 years.^23^ Previous studies have revealed seasonal variations in the onset of KD worldwide^8^^,^^9^; the seasonality of KD may be related to the exposure to environmental factors including unspecified infectious agents.^7^^,^^24^^,^^25^ These findings suggest that the combination of patient age at KD onset and sex, which are predisposing non-modifiable host factors, plays a considerable role and contributes to the influence of seasonal variations on environmental factors in the development of KD-related CAAs.

It is necessary to identify particular age groups of the same sex that may be at a higher risk of CAAs. This study primarily aimed to clarify the epidemiologic features of KD-related CAAs, based on consecutive KD cases that occurred in one area; this evaluation considered the seasons of the year in which KD occurred.

## METHODS

### Study population

As previously reported,^22^^,^^23^ we constructed a database of KD patients using data from our annual surveys conducted every October in pediatric departments across Wakayama Prefecture, Japan. According to the 2010 census data, this 4,726-km^2^ prefecture had 137,677 residents aged <15 years, including 37,249 residents aged <5 years (19,081 males, 18,168 females).^26^ In these surveys, pediatricians were asked to report all patients with KD using hospital medical records. In these surveys, the response rate from hospitals was 100%. The data collected from these surveys included patient age (in months) at disease onset, sex, date of disease onset, IVIG administration, and coronary artery findings. The presence/absence of CAAs was evaluated using transthoracic two-dimensional echocardiography (2-DE) 1 month after KD onset.

To consider the possibility of applying the study findings in clinical practice, patient age at disease onset was divided into five categories of age in months (<6, 6–11, 12–35, 36–59, and ≥60).^17^^,^^18^^,^^21^^,^^27^^,^^28^ Patient sex was referred to as males (1) and females (0). Four seasons were defined month-wise as spring (March, April, May), summer (June, July, August), autumn (September, October, November), and winter (December, January, February)^13^^,^^23^^,^^29^; these seasons are relevant to the climate in Japan.

The study protocol was approved by the ethics committee of Wakayama Medical University (Reference No. 794). This retrospective observational study provided participants the choice to opt-out; this was mentioned on the website of Wakayama Medical University.

### Survey period and study participants

All study participants were patients with KD identified between October 1, 1999, and September 30, 2017. No data have been excluded from the study.

### Diagnostic criteria of KD

The diagnosis of KD was made on the basis of the criteria from the 4th or 5th (since 2002) Diagnostic Guidelines established by the Japan Kawasaki Disease Research Committee,^30^^–^^32^ which were consistent with those used in Japanese nationwide surveys.^8^^,^^32^ The diagnostic criteria for KD consist of six principal symptoms as follows: prolonged fever lasting ≥5 days; bilateral non-purulent conjunctival injection; erythema of the oral mucosa, lips, and tongue; polymorphous skin rash; erythematous indurations of palm and soles; and non-purulent cervical lymphadenopathy. In the present study, the clinical diagnosis of KD included two diagnostic categories: “complete presentation” cases, diagnosed based on at least five items of the six principal symptoms, or four of those items when coronary aneurysm or dilatation was seen on 2-DE, and “incomplete presentation” cases, diagnosed based on four principal symptoms without coronary aneurysm or dilatation seen on 2-DE, or fewer principal symptoms with or without coronary aneurysm or dilatation seen on 2-DE.^8^^,^^31^

### Evaluation of CAAs

In this study, the primary outcome measure was the presence or absence of CAAs evaluated using the Japanese Ministry of Health (JMH) criteria.^33^ CAAs were defined as dilatation of a coronary artery (including aneurysm) or stenosis (including occlusion) detected by transthoracic 2D echocardiograms 1 month (around 30 days) after KD onset. As previously reported, these echocardiographic findings were collected as multiple sets of measurements by pediatric cardiologists and/or well-trained pediatricians and interpreted by pediatric cardiologists.^21^ In this study, CAAs including giant aneurysms, medium- or small-sized aneurysms, and dilatation were defined as follows: internal lumen diameter >3 mm in children <5 years old or >4 mm in children ≥5 years old, the internal diameter of a segment measures ≥1.5 times that of an adjacent segment, or clear irregularity in the coronary lumen.^33^^,^^34^ A lumen diameter of any coronary segment >8 mm was defined as a giant aneurysm; this was consistent with the criteria for echocardiography-based severity classification of coronary artery lesions related to KD.^34^^,^^35^

### Statistical analyses

First, we described the epidemiologic features of the onset of KD and the development of CAAs. Descriptive analyses of the study variables were then performed across the five categories based on age in months at disease onset. In this study, statistically significant differences in categorical variables were tested using the chi-square test.

The odds ratios (ORs) with 95% confidence intervals (CIs) of patient age (<6, 6–11, 12–35, 36–59, and ≥60) for the development of CAAs (relative to the values in patients aged 12–35 months, showing preponderance) were calculated using logistic regression models. Confounding factors were the sex and the climate season at disease onset. Owing to the efficacy of IVIG treatment in reducing the prevalence of KD-related coronary aneurysms,^1^^,^^8^^,^^14^^,^^35^ logistic regression models adjusted for the presence/absence of administration of first-line IVIG were used in this study. Sensitivity analyses were conducted using KD cases treated with first-line IVIG (eTable 1). To exclude any effects of sex, the ORs of patient age at disease onset for the development of CAAs were calculated using a similar model stratified by sex (eTable 2). Finally, the ORs with 95% CIs of the combination of patient age (five categories) and sex for the development of CAAs were calculated, stratified by different age/sex groups, and adjusted for four seasonal patterns of disease onset and for first-line IVIG administration (males aged 12–35 months comprised >50% of the sample and were used as the reference group).

Statistical analyses were performed using SPSS version 25 (IBM Corp., Armonk, NY, USA). Statistical significance was defined as a two-tailed *P*-value <0.05.

## RESULTS

Overall, the median age of 2,106 patients (1,215 males, 891 females; male-to-female ratio: 1.36) at disease onset was 25 (range, 1–212) months, and patients aged 12–35 months accounted for over half of the cohort. Table 1 shows the study variables across the five categories of patient age at KD onset. There was an inverse linear correlation between the proportion of males and increasing age; the older the age group, the lower the proportion of males. The highest age group (≥5 years [range 60–212 months]) had an inverse male-to-female ratio (Table 1). The proportion of KD occurrence in summer/autumn was higher in younger participants than in their older counterparts (56.9%, 43.9%, 42.8%, 41.5%, and 39.8% across increasing age categories) (Table 1). Therefore, the lowest age group (<6 months) showed an inverse summer/autumn to winter/spring ratio (>1.0).

**Table 1.** Characteristics of study participants stratified by age at onset of Kawasaki disease (KD) (*n* = 2,106)

	Total	Age at onset of KD, months					*P*-value^a^

		<6	6–11	12–35	36–59	≥60	
Participants, *n*	2,106	160	326	909	460	251	
Male, *n* (%)	1,215 (57.7)	105 (65.6)	203 (62.3)	528 (58.1)	255 (55.4)	124 (49.4)	0.005
Season at disease onset, *n* (%)							
Spring (March, April, May)	517 (24.5)	24 (15.0)	80 (24.5)	219 (24.1)	131 (28.5)	63 (25.1)	0.028
Summer (June, July, August)	510 (24.2)	54 (33.8)	85 (26.1)	204 (22.4)	112 (24.3)	55 (21.9)	
Autumn (September, October, November)	404 (19.2)	37 (23.1)	58 (17.8)	185 (20.4)	79 (17.2)	45 (17.9)	
Winter (December, January, February)	675 (32.1)	45 (28.1)	103 (31.6)	301 (33.1)	138 (30.0)	88 (35.1)	

Administration of intravenous immunoglobulin (IVIG), *n* (%)	1,963 (93.2)	148 (92.5)	298 (91.4)	854 (93.9)	432 (93.9)	231 (92.0)	0.480
IVIG non-responders, *n* (%)	446 (22.7)	30 (20.3)	57 (19.1)	198 (23.2)	93 (21.5)	68 (29.4)	0.059
Male, *n* (%)	302 (67.7)	22 (73.3)	40 (70.2)	137 (69.2)	61 (65.6)	42 (61.8)	0.721
Season at disease onset, *n* (%)							
Spring (March, April, May)	129 (28.9)	5	15	50	41	18	0.006
Summer (June, July, August)	85 (19.1)	11	13	37	10	14	
Autumn (September, October, November)	86 (19.3)	5	6	41	15	19	
Winter (December, January, February)	146 (32.7)	9	23	70	27	17	

^a^*P*-values from chi-square test.

The proportion of patients with CAAs was 2.8% (3.4% of males, 2.1% of females; *P* = 0.091), and the proportion of patients with CAAs was significantly higher in those not receiving IVIG treatment than in those who received it (5.6% (8/143) vs 2.6% (52/1,963); *P* = 0.047). A U-shaped tendency with the 1- to 2-year age group in the bottom was observed across the five age categories (Table 2). With regard to patients with CAAs, 75% (6/8) of those who developed giant aneurysms were females; 75% (39/52) of those who developed medium-sized or smaller aneurysms were males (Table 2). The proportion of patients who developed CAAs was significantly lower during summer months than in other seasons (1.6% vs 3.3%; *P* = 0.047). In patients with giant aneurysms, 50% (4/8) experienced KD occurrence in autumn (*P* = 0.049); however, this season had the lowest proportion of patients with KD (19.2%).

**Table 2.** Characteristics of patients with coronary artery abnormalities stratified by age at onset of Kawasaki disease (KD) (*n* = 2,106)

	Total	Age at onset of KD, months					*P*-value^a^

		<6	6–11	12–35	36–59	≥60	
	(*n* = 2,106)	(*n* = 160)	(*n* = 326)	(*n* = 909)	(*n* = 460)	(*n* = 251)	
Coronary artery abnormalities, *n* (%)	60 (2.8)	8 (5.0)	10 (3.1)	15 (1.7)	15 (3.3)	12 (4.8)	0.025
Male/Female	41/19	4/4	6/4	12/3	11/4	8/4	0.610
Season at disease onset, *n* (%)							
Spring (March, April, May)	17 (28.3)	5	1	4	4	3	0.410
Summer (June, July, August)	8 (13.3)	1	2	3	0	2	
Autumn (September, October, November)	13 (21.7)	1	1	4	4	3	
Winter (December, January, February)	22 (36.7)	1	6	4	7	4	

Giant aneurysms, *n*	8	2	0	2	2	2	0.628
Male/Female	2/6	0/2	0	0/2	2/0	0/2	0.046
Season at disease onset, *n*							
Spring (March, April, May)	2	1	0	1	0	0	0.350
Summer (June, July, August)	1	1	0	0	0	0	
Autumn (September, October, November)	4	0	0	1	1	2	
Winter (December, January, February)	1	0	0	0	1	0	

^a^*P*-values from chi-square test.

As shown in Table 3, the adjusted ORs for CAAs among those aged <6 months (3.3; 95%, CI 1.4–8.0; *P* < 0.008) and ≥60 months (3.1; 95%, CI 1.4–6.7; *P* < 0.005) were significantly higher than among those aged 12–35 months. The ORs of males for the development of CAAs were likely to be higher than that of females (*P* = 0.069). The ORs of summer-onset disease for the development of CAAs were likely to be lower than those for winter-onset disease (*P* = 0.056). The ORs of missing IVIG therapy for the development of CAAs were higher than those for IVIG administration (*P* = 0.039). The results from the sensitivity analyses using 1,963 cases treated with first-line IVIG were similar (eTable 1).

**Table 3.** Odds ratios for coronary artery abnormalities (CAAs) at 1 month after the onset of Kawasaki disease (*n* = 2,106)

	Population at risk, *n* (%)	Patients with/without CAAs, *n* (%)	Crude			Adjusted^a^		
	
			OR	(95% CI)	*P*-value	OR	(95% CI)	*P*-value
Patient age at disease onset, months								
<6	160 (7.6)	8 (5.0)/152 (95.0)	3.14	(1.31, 7.53)	0.010	3.29	(1.36, 7.95)	0.008
6–11	326 (15.5)	10 (3.1)/316 (96.9)	1.89	(0.84, 4.24)	0.125	1.85	(0.82, 4.17)	0.139
12–35	909 (43.2)	15 (1.7)/894 (98.3)	1.00	reference		1.00	reference	
36–59	460 (21.8)	15 (3.3)/445 (96.7)	2.01	(0.97, 4.15)	0.059	2.06	(0.99, 4.26)	0.052
≥60	251 (11.9)	12 (4.8)/239 (95.2)	2.99	(1.38, 6.48)	0.005	3.07	(1.41, 6.69)	0.005
Patient sex								
Male	1,215 (57.7)	41 (3.4)/1,174 (96.6)	1.60	(0.92, 2.78)	0.093	1.68	(0.96, 2.93)	0.069
Female	891 (42.3)	19 (2.1)/872 (97.9)	1.00	reference		1.00	reference	
Season at disease onset								
Spring	517 (24.5)	17 (3.3)/500 (96.7)	1.01	(0.53, 1.92)	0.978	1.05	(0.55, 2.02)	0.874
Summer	510 (24.2)	8 (1.6)/502 (98.4)	0.47	(0.21, 1.07)	0.073	0.45	(0.20, 1.02)	0.056
Autumn	404 (19.2)	13 (3.2)/391 (96.8)	0.99	(0.49, 1.98)	0.970	1.00	(0.49, 2.01)	0.988
Winter	675 (32.1)	22 (3.3)/653 (96.7)	1.00	reference		1.00	reference	
Administration of intravenous immunoglobulin therapy								
Yes	1,963 (93.2)	52 (2.6)/1,911 (97.4)	1.00	reference		1.00	reference	
No	143 (6.8)	8 (5.6)/135 (94.4)	2.18	(1.01, 4.68)	0.046	2.26	(1.04, 4.91)	0.039

CI, confidence interval; OR, odds ratio.

^a^Adjusted for all variables listed in this table.

Using a similar model for the development of CAAs stratified by sex, the ORs of patient age at disease onset were significantly higher; however, the CIs were wide in males aged ≥60 months and in females aged <6 months (eTable 2). Table 4 shows the ORs of combined age and sex of KD patients for the development of CAAs. The ORs for CAAs adjusted for four seasons and presence/absence of first-line IVIG administration were significantly higher among males aged ≥60 months (3.0; 95%, CI 1.2–7.5; *P* = 0.021) and females aged <6 months (3.6; 95%, CI 1.1–11.8; *P* = 0.032).

**Table 4.** Odds ratios of patient age and sex for developing coronary artery abnormalities (CAAs) at 1 month after the onset of Kawasaki disease (*n* = 2,106)

	Age, months	Population at risk, *n* (%)	Patients with/without CAAs, *n* (%)	Crude			Adjusted^a^		
	
				OR	(95% CI)	*P*-value	OR	(95% CI)	*P*-value
Male	<6	105 (5.0)	4 (3.8)/101 (96.2)	1.70	(0.54, 5.39)	0.365	1.85	(0.58, 5.89)	0.299
	6–11	203 (9.6)	6 (3.0)/197 (97.0)	1.31	(0.49, 3.54)	0.595	1.32	(0.49, 3.56)	0.590
	12–35	528 (25.1)	12 (2.3)/516 (97.7)	1.00	reference		1.00	reference	
	36–59	255 (12.1)	11 (4.3)/244 (95.7)	1.94	(0.84, 4.46)	0.119	1.98	(0.86, 4.56)	0.109
	≥60	124 (5.9)	8 (6.5)/116 (93.5)	2.97	(1.19, 7.42)	0.020	2.97	(1.18, 7.45)	0.021

Female	<6	55 (2.6)	4 (7.3)/51 (92.7)	3.37	(1.05, 10.84)	0.041	3.63	(1.12, 11.76)	0.032
	6–11	123 (5.8)	4 (3.3)/119 (96.7)	1.45	(0.46, 4.56)	0.530	1.43	(0.45, 4.53)	0.547
	12–35	381 (18.1)	3 (0.8)/378 (99.2)	0.34	(0.10, 1.22)	0.098	0.34	(0.10, 1.22)	0.097
	36–59	205 (9.7)	4 (2.0)/201 (98.0)	0.86	(0.27, 2.68)	0.789	0.84	(0.27, 2.66)	0.771
	≥60	127 (6.0)	4 (3.1)/123 (96.9)	1.40	(0.44, 4.41)	0.567	1.33	(0.42, 4.22)	0.626

CI, confidence interval; OR, odds ratio.

^a^Adjusted for climate season (four categories) of onset and administration of intravenous immunoglobulin therapy.

## DISCUSSION

In this study, we clarified the epidemiologic features of KD-related CAAs, focusing on seasonal variations, and on whether the interaction between patient age at disease onset and sex influenced the development of CAAs at 1 month after disease onset. There were two major findings. First, after adjusting for the season in which KD occurred, incorporating patient sex into the analysis greatly increased the ORs, with females aged <6 months (youngest age category with the highest in male-to-female ratio) and males aged ≥5 years (oldest age category with the lowest in male-to-female ratio) having significantly increased odds of developing CAAs. These findings are consistent with those of previous epidemiological studies using Japanese nationwide KD surveys; both younger and older age are associated with risks of cardiac sequelae^17^^,^^18^; in cases of giant coronary aneurysms, patients aged <1 and ≥5 years are vulnerable.^28^ The present study has little selection bias with no missing data from 18-year consecutive KD cases that occurred in one area. To our knowledge, after incorporating seasonal variation of KD onset, this study was the first to identify sex-specific age groups that are at a high risk for developing KD-related CAAs.

Second, we found a specific relationship between the season in which KD occurred and the proportion of patients who developed CAAs including giant aneurysms. While the overall proportion of patients with CAAs were significantly lower in summer than in other seasons, the proportion of patients with giant aneurysms showed an increasing trend in autumn, with nadir incidence of KD occurrence. To the best of our knowledge, this may be a novel finding. Although the number of cases with giant aneurysms was inadequate for statistically testing seasonal variations in the development of CAAs, this study has little selection bias, as all consecutive KD cases with no missing data within an 18-year period were analyzed. There have been few previous studies focusing on the seasonal variation in KD-related CAAs. A six-hospital collaborating study in a single metropolitan region in Northern Kyushu region in Japan reported no observed seasonality in the distribution of patients with CAAs; however, seasonal variation was observed in the distribution of patients who were non-responsive to initial IVIG therapy, with a higher proportion of IVIG non-responders during the warm than the cold periods.^36^ In terms of the response to IVIG therapy in the acute-phase KD, a study using data from Japanese nationwide KD surveys (2009–2016; that have ∼75% response rate) showed differences between IVIG non-responders and responders in the seasonal periodicity calculated by Fourier analysis. The proportion of IVIG non-responders showed a decreasing trend in autumn and that of IVIG responders showed an increasing trend over autumn to winter.^29^ In this cohort, the seasonality of IVIG non-responders tended to differ by sex; the proportions of female IVIG non-responders tended to increase during summer, whereas those of their male counterparts tended to decrease in autumn.^29^ Those responses to IVIG therapy may reflect the etiological agents of KD and/or immune reactivity against such agents, which possibly differ by sex. Considering the robust evidence regarding the efficacy of IVIG therapy in reducing the prevalence of KD-related CAAs, studies for identifying the seasonal aspects of KD, particularly those of related CAAs, are lacking.

The mechanisms for the age-related risk of developing CAAs in KD patients have not been resolved. Essentially, our data demonstrate a specific association between patient age and the season of occurrence (>60% of patients who were younger than 4 months of age developed KD in summer and autumn).^23^ The seasonality of KD may be related to various infectious agents that act as a trigger in susceptible children.^7^^,^^8^^,^^10^^–^^12^^,^^23^^–^^25^^,^^29^ The etiology of KD onset is thought to involve gene–environmental interactions^7^^,^^37^ and patients affected at ages younger or older than the peak age of onset may also have characteristic genetic factors.^37^ More recently, an age-stratified genome-wide association study targeting both Korean and Japanese populations suspected a rare non-synonymous SNP (rs4365796) in the lymphoid enhancer binding factor 1 gene to be a susceptibility gene to specifically affect KD patients younger than 6 months of age.^38^ Interestingly, Biezeveld et al suggested that polymorphisms in the mannose-binding lectin gene are one of the determinants of age-defined risks of CAAs in KD patients, being protective in infants but potentially harmful in patients aged >1 year.^39^ Moreover, the clinically heterogeneous features of KD (eg, polymorphous skin rash, cervical lymphadenopathy, and abdominal signs) may be a clue to explore any candidate agents and/or heterogeneous gene-environment interactions modified by patient age.

The present findings suggest that sex-related vulnerability not only in the susceptibility to KD onset but also in development of CAAs varies according to age. The mechanism of sex-related risks for developing CAAs, as well as the susceptibility to KD, remains unresolved.^7^^,^^37^ Previous epidemiological studies reported that males were vulnerable in terms of their susceptibility to KD onset^7^^–^^9^^,^^20^ and the development of CAAs.^15^^,^^17^^–^^20^ More recently found sex-specific genetic variants involved in KD pathogenesis (eg, FCGR2A His167Arg polymorphism) may provide new insight into KD susceptibility.^40^ In conjunction with patients’ demographic data including combined age and sex, genomic data analyses stratified with coronary artery outcomes may be more efficient.

This study has all the limitations inherent to retrospective studies. In addition, the study population was limited to one geographical area. This study examined consecutive cases collated over an 18-year period in one prefecture in Japan (similar to a state in the USA); therefore, the findings were based on the data from a study population comprising Japanese patients with KD and cannot be directly applied to those of different racial or ethnic backgrounds. However, the incidence of KD in Japan is extremely high^8^^,^^9^; thus, the findings from this study could be used as a representative or reference sample. The definition of CAAs in the present study remains a major limitation. The use of JMH criteria^33^^,^^35^ makes generalization to KD populations evaluated using Z scores particularly difficult. Compared with the American Heart Association (AHA) Z-score criteria, the JMH criteria have recently been shown to underestimate the severity of aneurysms in the youngest age cohort.^41^ We also agree that the AHA Z-score criteria are clinically useful in detecting coronary artery dilatation at the point of diagnosis.^42^ In comparison with the AHA Z-score criteria, the present definition of CAAs developing 1 month after KD onset based on JMH criteria may underestimate the severity of aneurysms in the youngest age cohort. However, this becomes more important as the present study found an increased risk of CAAs in females aged <6 months. Moreover, the present epidemiological figures regarding giant aneurysms are based on a limited number of cases. In view of the small sample size, these findings should be interpreted with caution. The present study provides a detailed descriptive epidemiology with the actual number of cases.

In conclusion, this study clarified the vulnerability of younger female and older males for developing KD-related CAAs 1 month after disease onset, by incorporating the seasonal variations of KD occurrence. These findings were based on consecutive cases collated over an 18-year period in one prefecture in Japan. Moreover, using the same data with little selection bias, we examined the most vulnerable population (the combination of patient age and sex) in terms of the development of KD-related CAAs. Our findings suggest that the seasonality of KD occurrence and stratification of patients based on age and sex are helpful both in exploring environmental factor(s), including infectious agents as a trigger of KD occurrence, and for developing more appropriate treatment strategies for acute-phase KD.

## APPENDIX A. SUPPLEMENTARY DATA

eTable 1. Odds ratios for coronary artery abnormalities (CAAs) at 1 month after disease onset among Kawasaki disease cases treated with first-line intravenous immunoglobulin (*n* = 1,963)

eTable 2. Odds ratios of patient age for developing coronary artery abnormalities (CAAs) at 1 month after the onset of Kawasaki disease, stratified by sex (*n* = 2,106)

## References

[1] McCrindle BW, Rowley AH, Newburger JW, ; American Heart Association Rheumatic Fever, Endocarditis, and Kawasaki Disease Committee of the Council on Cardiovascular Disease in the Young; Council on Cardiovascular and Stroke Nursing; Council on Cardiovascular Surgery and Anesthesia; Council on Epidemiology and Prevention Diagnosis, treatment, and long-term management of Kawasaki disease: a scientific statement for health professionals from the American Heart Association. Circulation. 2017;135:e927–e999. Correction to: Diagnosis, Treatment, and Long-Term Management of Kawasaki Disease: A Scientific Statement for Health Professionals From the American Heart Association. *Circulation*. 2019;140:e181–e184. 10.1161/CIR.0000000000000484 28356445

[2] Takahashi K, Oharaseki T, Yokouchi Y Histopathological aspects of cardiovascular lesions in Kawasaki disease. Int J Rheum Dis. 2018;21:31–35. 10.1111/1756-185X.13207 29105353

[3] Kato H, Sugimura T, Akagi T, Long-term consequences of Kawasaki disease. A 10- to 21-year follow-up study of 594 patients. Circulation. 1996;94:1379–1385. 10.1161/01.CIR.94.6.1379 8822996

[4] Fukazawa R, Kobayashi T, Mikami M, Nationwide survey of patients with giant coronary aneurysm secondary to Kawasaki disease 1999–2010 in Japan. Circ J. 2017;82:239–246. 10.1253/circj.CJ-17-0433 28855435

[5] Daniels LB, Tjajadi MS, Walford HH, Prevalence of Kawasaki disease in young adults with suspected myocardial ischemia. Circulation. 2012;125:2447–2453. 10.1161/CIRCULATIONAHA.111.082107 22595319 PMC3393523

[6] Kawasaki T Acute febrile mucocutaneous syndrome with lymphoid involvement with specific desquamation of the fingers and toes in children. Jpn J Allergy. 1967;16:178–222. 6062087

[7] Nakamura Y Kawasaki disease: epidemiology and the lessons from it. Int J Rheum Dis. 2018;21:16–19. 10.1111/1756-185X.13211 29115029

[8] Makino N, Nakamura Y, Yashiro M, Epidemiological observations of Kawasaki disease in Japan, 2013–2014. Pediatr Int. 2018;60:581–587. 10.1111/ped.13544 29498791

[9] Uehara R, Belay ED Epidemiology of Kawasaki disease in Asia, Europe, and the United States. J Epidemiol. 2012;22:79–85. 10.2188/jea.JE20110131 22307434 PMC3798585

[10] Ozeki Y, Yamada F, Saito A, Epidemiologic features of Kawasaki disease distinguished by seasonal variation: an age-specific analysis. Ann Epidemiol. 2018;28:796–800. 10.1016/j.annepidem.2018.08.004 30181018

[11] Sano T, Makino N, Aoyama Y, Temporal and geographical clustering of Kawasaki disease in Japan: 2007–2012. Pediatr Int. 2016;58:1140–1145. 10.1111/ped.12970 26940079

[12] Burns JC, Cayan DR, Tong G, Seasonality and temporal clustering of Kawasaki syndrome. Epidemiology. 2005;16:220–225. 10.1097/01.ede.0000152901.06689.d4 15703537 PMC2894624

[13] Abrams JY, Blase JL, Belay ED, Increased Kawasaki disease incidence associated with higher precipitation and lower temperatures, Japan, 1991–2004. Pediatr Infect Dis J. 2018;37:526–530. 10.1097/INF.0000000000001838 29140936

[14] Newburger JW, Takahashi M, Beiser AS, A single intravenous infusion of gamma globulin as compared with four infusions in the treatment of acute Kawasaki syndrome. N Engl J Med. 1991;324:1633–1639. 10.1056/NEJM199106063242305 1709446

[15] Yanagawa H, Tuohong Z, Oki I, Effects of gamma-globulin on the cardiac sequelae of Kawasaki disease. Pediatr Cardiol. 1999;20:248–251. 10.1007/s002469900458 10368448

[16] Research Committee of the Japanese Society of Pediatric Cardiology; Cardiac Surgery Committee for Development of Guidelines for Medical Treatment of Acute Kawasaki Disease Guidelines for medical treatment of acute Kawasaki disease: report of the Research Committee of the Japanese Society of Pediatric Cardiology and Cardiac Surgery (2012 revised version). Pediatr Int. 2014;56:135–158. 10.1111/ped.12317 24730626

[17] Nakamura Y, Fujita Y, Nagai M, Cardiac sequelae of Kawasaki disease in Japan: statistical analysis. Pediatrics. 1991;88:1144–1147. 1720235

[18] Hirose K, Nakamura Y, Yanagawa H Cardiac sequelae of Kawasaki disease in Japan over 10 years. Acta Paediatr Jpn. 1995;37:667–671. 10.1111/j.1442-200X.1995.tb03401.x 8775548

[19] Zhang T, Yanagawa H, Oki I, Nakamura Y Factors relating to the cardiac sequelae of Kawasaki disease one month after initial onset. Acta Paediatr. 2002;91:517–520. 10.1111/j.1651-2227.2002.tb03270.x 12113319

[20] Belay ED, Maddox RA, Holman RC, Curns AT, Ballah K, Schonberger LB Kawasaki syndrome and risk factors for coronary artery abnormalities: United States, 1994–2003. Pediatr Infect Dis J. 2006;25:245–249. 10.1097/01.inf.0000202068.30956.16 16511388

[21] Muta H, Ishii M, Sakaue T, Older age is a risk factor for the development of cardiovascular sequelae in Kawasaki disease. Pediatrics. 2004;114:751–754. 10.1542/peds.2003-0118-F 15342849

[22] Kitano N, Suzuki H, Takeuchi T, ; Wakayama Kawasaki Disease Study Group Epidemiologic features and prognostic factors of coronary artery lesions associated with Kawasaki disease based on a 13-year cohort of consecutive cases identified by complete enumeration surveys in Wakayama, Japan. J Epidemiol. 2014;24:427–434. 10.2188/jea.JE20140018 24998951 PMC4150015

[23] Kitano N, Suzuki H, Takeuchi T Patient age and the seasonal pattern of onset of Kawasaki’s disease. N Engl J Med. 2018;378:2048–2049. 10.1056/NEJMc1804312 29791820

[24] Burns JC, Glodé MP Kawasaki syndrome. Lancet. 2004;364:533–544. 10.1016/S0140-6736(04)16814-1 15302199

[25] Rowley AH Is Kawasaki disease an infectious disorder? Int J Rheum Dis. 2018;21:20–25. 10.1111/1756-185X.13213 29105346 PMC5777874

[26] Statistics and Information Department, Minister’s Secretariat, Ministry of Health, Labour and Welfare. *Vital statistics of Japan, 2010 volume 1*. Tokyo: Health and Welfare Statistics Association; 2012.

[27] Chang FY, Hwang B, Chen SJ, Lee PC, Meng CC, Lu JH Characteristics of Kawasaki disease in infants younger than six months of age. Pediatr Infect Dis J. 2006;25:241–244. 10.1097/01.inf.0000202067.50975.90 16511387

[28] Sudo D, Monobe Y, Yashiro M, Sadakane A, Uehara R, Nakamura Y Case-control study of giant coronary aneurysms due to Kawasaki disease: the 19th nationwide survey. Pediatr Int. 2010;52:790–794. 10.1111/j.1442-200X.2010.03161.x 20487371

[29] Kido S, Ae R, Kosami K, Seasonality differs by IVIG responsiveness in patients with Kawasaki disease. Pediatr Int. 2019;61:539–543. 10.1111/ped.13863 30980447

[30] Japan Kawasaki Disease Research Committee. *Diagnostic guidelines of Kawasaki disease* [in Japanese], 4th ed. Tokyo: The Japan Kawasaki Disease Research Committee; 1984.

[31] Ayusawa M, Sonobe T, Uemura S, Revision of diagnostic guidelines for Kawasaki disease (the 5th revised edition). Pediatr Int. 2005;47:232–234. 10.1111/j.1442-200x.2005.02033.x 15771703

[32] Yanagawa H, Kawasaki T, Shigematsu I Nationwide survey on Kawasaki disease in Japan. Pediatrics. 1987;80:58–62. 3601519

[33] Research Committee on Kawasaki Disease. *Report of Subcommittee on Standardization of Diagnostic Criteria and Reporting of Coronary Artery Lesions in Kawasaki disease* [in Japanese]. Tokyo, Japan: Ministry of Health and Welfare; 1984.

[34] Newburger JW, Takahashi M, Gerber MA, ; Committee on Rheumatic Fever, Endocarditis, and Kawasaki Disease, Council on Cardiovascular Disease in the Young, American Heart Association Diagnosis, treatment, and long-term management of Kawasaki disease: a scientific statement for health professionals from the Committee on Rheumatic Fever, Endocarditis, and Kawasaki Disease, Council on Cardiovascular Disease in the Young, American Heart Association. Pediatrics. 2004;114:1708–1733. 10.1542/peds.2004-2182 15574639

[35] Japanese Circulation Society Joint Research Group Guidelines for diagnosis and management of cardiovascular sequelae in Kawasaki disease. Pediatr Int. 2005;47:711–732. 10.1111/j.1442-200x.2005.02149.x 16354233

[36] Shimizu D, Hoshina T, Kawamura M, Seasonality in clinical courses of Kawasaki disease. Arch Dis Child. 2019;104:694–696. 10.1136/archdischild-2018-315267 30413486

[37] Onouchi Y The genetics of Kawasaki disease. Int J Rheum Dis. 2018;21:26–30. 10.1111/1756-185X.13218 29152908

[38] Kim HJ, Yun SW, Yu JJ, Identification of LEF1 as a susceptibility locus for Kawasaki disease in patients younger than 6 months of age. Genomics Inform. 2018;16:36–41. 10.5808/GI.2018.16.2.36 30304924 PMC6187808

[39] Biezeveld MH, Geissler J, Weverling GJ, Polymorphisms in the mannose-binding lectin gene as determinants of age-defined risk of coronary artery lesions in Kawasaki disease. Arthritis Rheum. 2006;54:369–376. 10.1002/art.21529 16385529

[40] Kwon YC, Kim JJ, Yun SW, ; Korean Kawasaki Disease Genetics Consortium Male-specific association of the FCGR2A His167Arg polymorphism with Kawasaki disease. PLoS One. 2017;12:e0184248. 10.1371/journal.pone.0184248 28886140 PMC5590908

[41] Burns JC, Hoshino S, Kobayashi T Kawasaki disease: an essential comparison of coronary artery aneurysm criteria. Lancet Child Adolesc Health. 2018;2:840–841. 10.1016/S2352-4642(18)30334-1 30337184

[42] Son MBF, Gauvreau K, Kim S, Predicting coronary artery aneurysms in Kawasaki disease at a North American Center: an assessment of baseline z scores. J Am Heart Assoc. 2017;6:e005378. 10.1161/JAHA.116.005378 28566299 PMC5669166

