# Supplementary material for: Seasonal Variation in Epidemiology of Kawasaki Disease-Related Coronary Artery Abnormalities in Japan, 1999–2017
Source: J Epidemiol. 2021 Feb 5;31(2):132–8. doi: 10.2188/jea.JE20190189 (PMC7813765; doi:10.2188/jea.JE20190189)
Supplement: Supplementary file 1 [file je-31-132-s001.pdf]

eTable 1. Odds ratios for coronary artery abnormalities (CAAs) at 1 month after disease onset among Kawasaki disease cases treated with first-line intravenous immunoglobulin (n=1,963)

	Population at risk, n (%)	Patients with/without CAAs, n (%)	Crude			Adjusted[a]		
			OR	(95% CI)	*P*-value	OR	(95% CI)	*P*-value
Patient age at disease onset, months								
<6	148 (7.5)	6 (4.1) / 142 (95.9)	2.73	(1.02, 7.31)	0.045	3.38	(1.21, 9.45)	0.020
6–11	298 (15.2)	7 (2.3) / 291 (97.7)	1.56	(0.62, 3.94)	0.351	1.86	(0.72, 4.83)	0.201
12–35	854 (43.5)	13 (1.5) / 841 (98.5)	1.00	reference		1.00	reference	
36–59	432 (22.0)	14 (3.2) / 418 (96.8)	2.17	(1.01, 4.65)	0.047	2.39	(1.09, 5.26)	0.030
≥60	231 (11.8)	12 (5.2) / 219 (94.8)	3.55	(1.60, 7.88)	0.002	3.26	(1.43, 7.46)	0.005
Patient sex								
Male	1,141 (58.1)	38 (3.3) / 1,103 (96.7)	1.99	(1.07, 3.69)	0.030	1.57	(0.82, 2.99)	0.170
Female	822 (41.9)	14 (1.7) / 808 (98.3)	1.00	reference		1.00	reference	
Season at disease onset								
Spring	489 (24.9)	15 (3.1) / 474 (96.9)	1.07	(0.53, 2.14)	0.851	0.93	(0.45, 1.92)	0.842
Summer	469 (23.9)	7 (1.5) / 462 (98.5)	0.51	(0.21, 1.24)	0.136	0.55	(0.22, 1.36)	0.192
Autumn	379 (19.3)	12 (3.2) / 367 (96.8)	1.10	(0.53, 2.32)	0.793	1.07	(0.49, 2.32)	0.872
Winter	626 (31.9)	18 (2.9) / 608 (97.1)	1.00	reference		1.00	reference	
Administration of second-line treatment								
Yes	1,963 (93.2)	52 (2.6) / 1,911 (97.4)	13.86	(7.06, 27.21)		12.96	(6.54, 25.68)	<0.001
No	143 (6.8)	8 (5.6) / 135 (94.4)	1.00	reference		1.00	reference	

CI, confidence interval; OR, odds ratio.

[a]Adjusted for all variables listed in this table.

eTable 2. Odds ratios of patient age for developing coronary artery abnormalities (CAAs) at 1 month after the onset of Kawasaki disease, stratified by sex (n=2,106)

**Male (n=1,215)**		Population at risk, n (%)	Patients with / without CAAs, n (%)	Crude			Adjusted[a]		
				OR	(95% CI)	*P*-value	OR	(95% CI)	*P*-value
Age, months	<6	105 (8.6)	4 (3.8) / 101 (96.2)	1.70	(0.54, 5.39)	0.365	1.87	(0.59, 5.95)	0.292
	6–11	203 (16.7)	6 (3.0) / 197 (97.0)	1.31	(0.49, 3.54)	0.595	1.33	(0.49, 3.61)	0.572
	12–35	528 (43.5)	12 (2.3) / 516 (97.7)	1.00	reference		1.00	reference	
	36–59	255 (21.0)	11 (4.3) / 244 (95.7)	1.94	(0.84, 4.46)	0.119	2.02	(0.88, 4.65)	0.099
	≥60	124 (10.2)	8 (6.5) / 116 (93.5)	2.97	(1.19, 7.42)	0.020	2.99	(1.19, 7.51)	0.019
**Female (n=891)**		Population at risk, n (%)	Patients with/without CAAs, n (%)	Crude			Adjusted[a]		
				OR	(95% CI)	*P*-value	OR	(95% CI)	*P*-value
Age, months	<6	55 (6.2)	4 (7.3) / 51 (92.7)	9.88	(2.15, 45.42)	0.003	11.32	(2.40, 53.35)	0.002
	6–11	123 (13.8)	4 (3.3) / 119 (96.7)	4.24	(0.94, 19.19)	0.061	3.95	(0.85, 18.24)	0.079
	12–35	381 (42.8)	3 (0.8) / 378 (99.2)	1.00	reference		1.00	reference	
	36–59	205 (23.0)	4 (2.0) / 201 (98.0)	2.51	(0.56, 11.31)	0.232	2.47	(0.54, 11.26)	0.244
	≥60	127 (14.3)	4 (3.1) / 123 (96.9)	4.10	(0.91, 18.56)	0.067	3.94	(0.86, 18.07)	0.078

CI, confidence interval; OR, odds ratio.

[a]Adjusted for climate season (four categories) of onset and administration of intravenous immunoglobulin therapy.
